# Blood mineral profile of French Alpine goats during the first third of the lactation period

**DOI:** 10.5194/aab-68-201-2025

**Published:** 2025-03-17

**Authors:** Zvonko Antunović, Željka Klir Šalavardić, Luka Zmaić, Josip Novoselec

**Affiliations:** 1 Department of Animal Production and Biotechnology, Faculty of Agrobiotechnical Sciences Osijek, University of J.J. Strossmayer in Osijek, V. Preloga 1, 31000 Osijek, Croatia; 2 Faculty of Medicine Osijek, University of Josip Juraj Strossmayer, J. Hutllera 4, 31000 Osijek, Croatia

## Abstract

The research objective of this study was to determine the blood mineral profile of French Alpine goats during the first third of the lactation period. The blood mineral profile refers to concentrations of macro-elements and micro-elements. The research was carried out on 20 5-year-old goats in their fourth lactation. Goats were fed forage feed (1.5 kg d^−1^). Alfalfa hay and water were available ad libitum during the experimental period. Concentrations of micro-elements in the blood and feed of French Alpine goats were determined by inductively coupled plasma mass spectrometry (ICP-MS). A comparison of measured average concentrations of macro-elements with the reference range for goats confirmed lower concentrations of K and Zn and higher concentrations of P, while concentrations of Na, Mn, and Se in this research were at the upper limit of the reference range. Such results can be explained by both the availability of the respective minerals in the goats' feed and the various reference ranges applied in the determination of the mineral profile of goats' blood, along with different laboratory methods used for blood mineral profiling. When analysing the time of sampling, the first third of the lactation period exhibited significantly lower concentrations of P, Mg, K, Fe, Zn, Mo, and Co in the goats' blood sampled in the period from the 30th day to the 90th day of lactation. Concentrations of Ca, Na, Cu, Mn, and Se in the goats' blood did not depend significantly on the time of sampling, although these were lower on the 90th day than on the 30th day of sampling. Significantly positive correlations were determined between the following mineral concentrations: Ca:Mg , Ca:K , Ca:Na , Ca:P , Ca:Zn , Ca:Se , Mg:K , Mg:Na , Mg:Cu , Mg:Fe , Mg:Zn , Mg:P , Mg:Mo , K:Na , K:Cu , K:Zn , K:P , K:Se , Na:Cu , Na:Zn , Na:P , Na:Se , Cu:P , Fe:Zn , Fe:Mn , Zn:P , and P:Se . The analysis of the measured concentrations of blood minerals and of correlations between them can be useful in the determination of the animals' health statuses during the first third of the lactation period; as such, this information indicates the need for feed supplementation or for the revision of the blood test reference range for specific goat breeds.

## 1 Introduction

Recently, goat farming has been gaining popularity on a global scale. As reported by the Food and Agriculture Organization (FAO), the global goat population is 1.002 billion, being most widespread in Asia (57.7 %) and Africa (35.7 %). The global goat population has doubled in the last 30 years (Lohani and Bhandari, 2021). If considering the period covering the past 4 decades, it has more than doubled (Utaaker et al., 2021). Considering our present circumstances with regard to climate change and its global effects, goat farming is gaining importance as goats emit less methane than other livestock, and so their influence on climate is acceptable (Darcan and Silanikove, 2018). Today, goat production comprises over 300 breeds of indigenous and commercial breeds and more than 1.006 million individuals (Ghanatsaman et al., 2023). The main goat produce is milk, which is especially valued in European goat production, and so carefully selected dairy goat breeds are dominant in Europe (Castro et al., 2023). The Alpine goat is one of the most widespread breeds of dairy goats in the world (Antunović et al., 2013). Following the global trends, goat farming in Croatia has also been growing lately, reaching more than 70 000 individuals. According to the report of the Croatian Agricultural Agency (CAA, 2023), out of the total number of goats within the selection (8190), Alpine goats constitute 54.1 %; i.e. 4427 goats are kept by 48 farmers. In a total of 3068 finished lactations, Alpine goats spent 243 d in lactation and produced, on average, 676 kg of milk, containing, on average, 3.2 % of milk fat and 3.1 % of protein (CAA, 2023). The high milk production of lactating Alpine goats can have a negative effect on the animal itself as lactation causes significant stress levels. During the lactation period, not only in its early stage but also in the first half of the total lactation period, lactating goats are exposed to demanding milk synthesis, following which they need to regain the body mass lost as a result, and so the production of milk has to be supported by high-quality feed (Antunović et al., 2018). However, many lactating goats are kept in inappropriate housing conditions and fed forage of poor quality, and so the metabolism of nutrients is often disturbed, leading to the occurrence of various diseases, primarily metabolic diseases. Blood mineral profiles of small ruminants during lactation may indicate feeding problems or show some clinical signs of disease if concentrations of minerals are marginally deficient (Pugh, 2023; Antunović et al., 2021). According to Hussein et al. (2022), the examination of the blood mineral profile in dairy cows, which includes elements that are poisonous and essential, can give basic knowledge to practitioners who monitor herds on a regular basis. There are many factors that influence the metabolism of minerals, such as quality of diets, nutritive composition of forage, genetic factors (sex, age, genotype), production status, stage of lactation, and laboratory procedures (Antunović et al., 2021; Samardžija et al., 2011; Mbassa and Poulsen, 1991). Changes in the concentrations of minerals in blood can depend on the absorption of minerals from the intestine and redistribution from endogenous stores (Piccione et al., 2007). In most animals, absorption of micro-elements occurs in the small intestine (Goff, 2018; Byrne and Murphy, 2022). However, in ruminants – and, especially, in the rumen – there are interactions between microorganisms, minerals, and other dietary substances which can result in the reduced absorption of minerals in intestines (Goff, 2018). These interactions can be caused by low solubility, as explained in the research by Vigh et al. (2023) on ruminal solubility of various micro-element sources which are used for ruminant supplementation and their effect on microbial populations and fermentation in rumen. Minerals are important in the animal organism: their actions are significantly intertwined, and they are indispensable for animal growth and production capacity (Marquès et al., 2022; Mayasula et al., 2021). Considering the above-mentioned facts, the determination of blood mineral profiles of lactating Alpine goats can be helpful to farmers to optimise feeding and increase milk yield. The blood mineral profile refers to concentrations of macro-elements and micro-elements. Macro-elements (Ca, P, Mg, Cl, K, Na, and S) are required in large quantities, and micro-elements (Fe, Zn, Cu, Se, Mn, Mo, and Co) are required in smaller quantities. Some studies deal with the determination of only several minerals in the blood of Alpine goats and some elaborate effects of lactation stage and sampling day during lactation (Antunović et al., 2009, 2017; Mezzetti et al., 2024); yet, there are not enough studies covering the wider scope of the mineral profile of Alpine goat blood and its changes under the influence of lactation stage. Since lactation, especially the first third of the lactation period, is a very demanding period for goats, this paper elaborates upon the changes that occur in the concentrations of minerals in the goats' blood.

## 2 Materials and method

### 2.1 Animals and diets

The research was carried out on French Alpine goats that were in the first third of the lactation period. There were 20 goats selected for the experiment, all being, on average, 5 years old and in their fourth lactation. The goats were selected from a herd of 50 goats; all were of good health and in good physical shape, and all had one offspring. The studied goats were kept on the Đurković family farm in Marjančaci, near Valpovo, in Osijek-Baranja County. This family farm is experienced in goat farming. The goats were tested on the 30th and 90th days of lactation. Goats were fed feed with alfalfa hay ad libitum, while feed mixture, based on 16 % crude protein, was offered to goats at an amount of 1.5 kg d^−1^, as recommended by the NRC (2007). Freshwater was offered to goats ad libitum. The feed mixture was comprised of corn (53 %), oats (10 %), wheat flour (4.6 %), soybean meal (19 %), soybean hulls (10 %), salt (0.4 %), and mineral vitamin premix (3 %). The chemical composition of the feed mixture was as follows: 89 % of dry matter (DM), 16 % of crude protein, 52.5 % of crude fibre, 5 % of crude ash, 2.9 % of crude lipids, and 11.3 MJ kg^−1^ DM of metabolisable energy. Milk yield was determined following the morning milking on the 30th and 90th days of lactation, amounting to 1.69 and 1.29 L, respectively. Following this, the milk chemical composition was determined: 3.23 % of fat, 2.97 % of protein, 4.41 % of lactose, 8.44 % of non-fat dry matter, 21.82 mg dL^−1^ of urea, 5.58 log of somatic cell count, and 4.04 log of bacterial-colony-forming units on the 30th day and 3.25 % of fat, 2.93 % of protein, 4.29 % of lactose, 8.28 % of non-fat dry matter, 20.42 mg dL^−1^ of urea, 5.96 log of somatic cell count, and 4.22 log of bacterial-colony-forming units on the 90th day of lactation. This study was implemented within a wider research project related to the milk quality of French Alpine goats already published by Antunović et al. (2024).

### 2.2 Blood and feed analysis

**Figure 1:**
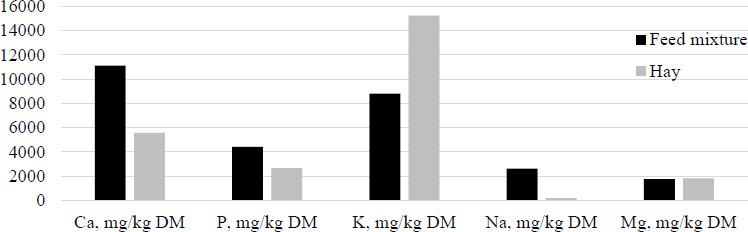
Concentration of minerals in goats' feed (DM – dry matter).

**Figure 2:**
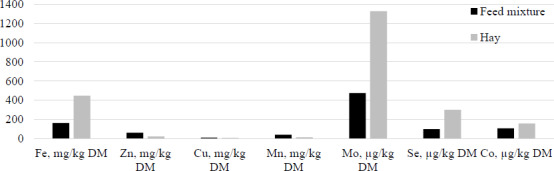
Concentration of micro-elements in goats' feed (DM – dry matter).

Blood was sampled from the same goats on two sampling occasions: before morning feeding and before milking. Blood samples were immediately transported to the laboratory of the Faculty of Agrobiotechnical Sciences Osijek. Samples were centrifuged to separate the serum, which was frozen at -80 °C. After defrosting, the sample was tested for concentrations of minerals, including macro-elements (Ca, P, K, Na, and Mg) and micro-elements (Fe, Zn, Cu, Se, Mn, Mo, and Co). Concentrations of micro-elements in the blood and feed of French Alpine goats were determined in inductively coupled plasma mass spectrometry (ICP-MS) by means of a coupled continuous mass spectrometer. Concentrations of minerals were determined in the feed of Alpine goats, as presented in Figs. 1 and 2.

### 2.3 Statistical analyses

The mean and standard deviation of blood minerals in the French Alpine goats were processed by means of the MEANS procedure, while the influence of the sampling day on the blood mineral profile was analysed by means of the generalised linear model (GLM) procedure and processed by the SAS 9.4^®^ (2002–2012). The following formula was used: $Y_{ijk}=\mu +s_{i}+e_{ij}$ , where μ is the overall mean, $s_{i}$ is the fixed effect of sampling day, and $e_{ij}$ is the random error variation. A comparison between the mean values of different groups was conducted using Tukey's test ( P<0.05 ). Correlations among minerals in the goats' blood were evaluated by means of Pearson's correlation (CORR procedure) and were declared to be significant if P<0.05 .

**Table 1:** Descriptive statistics of macro-elements of Alpine goats' serum during the first third of the lactation period.

Macro-element, mg L^−1^	Mean	SD	Minimum	Maximum	Reference value
Ca	85.32	13.49	65.22	141.26	83.6–110.00^a^
P	168.42	33.04	113.70	315.30	40–112^b^
K	165.29	24.74	139.36	275.18	186–272^a^
Na	3347.98	403.20	2829.99	5547.00	3220–3335^c^
Mg	22.22	3.86	13.87	37.82	19.4–26.7^d^

SD: standard deviation; ^a^ Schweinzer et al. (2017); ^b^ Jackson and Cockcroft (2002); ^c^ Underwood and Suttle (1999) for sheep; ^d^ Herdt and Hoff (2011).

**Table 2:** Descriptive statistics of micro-elements of Alpine goats' serum during the first third of the lactation period.

Micro-elements, mg L^−1^	Mean	SD	Minimum	Maximum	Reference value^∗^
Fe	1.35	1.04	0.10	5.65	1.05–2.71
Zn	0.64	0.10	0.45	0.90	0.27–1.09
Cu	0.96	0.50	0.13	2.30	0.50–1.20
Mn, $µg\;L^{-1}$	5.15	6.04	0.13	31.25	1.50–5.00
Se, $µg\;L^{-1}$	101.17	15.34	73.41	146.40	50.90–99.70
Mo, $µg\;L^{-1}$	13.71	6.52	3.92	31.74	2.20–219.00
Co, $µg\;L^{-1}$	1.36	0.67	0.25	2.94	0.20–1.60

SD: standard deviation; ^∗^ Schweinzer et al. (2017).

## 3 Results

Measured concentrations of minerals in the goats' blood (both macro-elements and micro-elements) were mostly similar to the concentrations determined by previous research (Tables 1 and 2). The comparison of measured average concentrations of macro-elements and micro-elements in the goats' blood with a reference range showed lower concentrations of K and higher concentrations of P, while concentrations of Na, Mn, and Se were close to the upper limits of the reference range. It was also proven that the concentrations of micro-elements had greater variability, as indicated by the established high standard deviations (Cu: 0.50; Mn: 6.04; Fe: 1.04).

**Table 3:** Effect of sampling day on macro-elements in Alpine goats' serum during the first third of the lactation period.

Mineral,	Sampling day		SEM	P value
mg L^−1^	30th day	90th day		
Ca	89.10	81.54	2.13	0.076
P	181.73	155.11	5.22	0.009
K	177.24	153.33	3.9	0.001
Na	3428.27	3267.70	63.75	0.212
Mg	23.77	20.66	0.61	0.009

SEM: standard error of means.

**Table 4:** Effect of sampling day on micro-elements of Alpine goats' serum during the first third of the lactation period.

Mineral,	Sampling day		SEM	P value
mg L^−1^	30th day	90th day		
Fe	1.70	1.00	0.16	0.032
Zn	0.67	0.60	0.01	0.031
Cu	1.04	0.88	0.08	0.309
Mn, $µg\;L^{-1}$	6.19	3.26	1.14	0.225
Se, $µg\;L^{-1}$	104.00	98.34	2.43	0.248
Mo, $µg\;L^{-1}$	15.81	11.60	1.03	0.040
Co, $µg\;L^{-1}$	1.60	1.12	0.11	0.024

SEM: standard error of means.

The sampling day during the first third of the lactation period (Tables 3 and 4) influenced the Alpine goats' serum as there were significantly lower concentrations of P, Mg, K, Fe, Zn, Mo, and Co in the blood on the 90th day compared to on the 30th day of lactation. Concentrations of Ca, Na, Cu, Mn, and Se in the goats' blood did not vary significantly with respect to the sampling day, although they were also lower on the 90th day than on the 30th day of lactation.

**Table 5:**
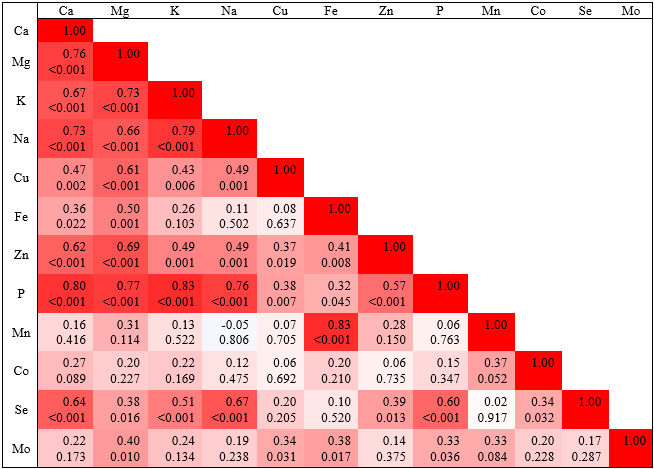
Correlation coefficients between concentrations of minerals in Alpine goats' serum during the first third of the lactation period.

The analysis of the correlation coefficients between the concentrations of measured minerals indicated many significant correlations ( P<0.01 ; Table 5). Significantly positive correlations were determined between Ca:Mg , Ca:K , Ca:Na , Ca:P , Ca:Zn , Ca:Se , Mg:K , Mg:Na , Mg:Cu , Mg:Fe , Mg:Zn , Mg:P , Mg:Mo , K:Na , K:Cu , K:Zn , K:P , K:Se , Na:Cu , Na:Zn , Na:P , Na:Se , Cu:P , Fe:Zn , Fe:Mn , Zn:P , and P:Se .

## 4 Discussion

Determining the mineral profile in animal blood (macro-elements and micro-elements) is important as it provides information on the supply of minerals through feed and on animals' health conditions. Blood sampling is a minimally invasive procedure (Herdt and Hoff, 2011). Filho et al. (2024) pointed out that the requirements for minerals in lactating goats of the Alpine breed depended significantly on concentrations of Mg and Na ( p=0.0211 and p=0.017 , respectively) only. Besides this, the requirements for macro-elements from feed decreased significantly ( P<0.05 ) after the 56th day of lactation, except for the requirements for total P and K. In their conclusion, the above-mentioned authors stated that Alpine goats in early lactation mobilised, on average, 12.1±0.3 g of Ca, 5.7±0.2 g of P, 0.9±0.1 g of Mg, 9.1±0.2 g of K, and 3.4±0.1 g of Na from empty body weight (EBW) per day. Furthermore, the total net requirements of Ca, P, Mg, K, and Na were 4.2±0.8 , 2.3±0.4 , 1.2±0.3 , 7.2±0.9 , and 1.1±0.2 g kg^−1^ milk, respectively, during the first 56 d of lactation. In our research, the determined average concentration of Ca in the goats' serum was slightly higher than the lower referential value, while the concentration of K was lower than the reference range, and the concentration of Na was higher than the reference range (Table 1). This indicated that the goats were provided with those macro-elements through feed (Fig. 1).

The determined concentrations of the most micro-elements in the serum of Alpine goats were within the reference range, except for the concentrations of Mn and Se, which were at the upper limit of the reference range. This is connected with the high-quality supply of minerals in feed (Fig. 2). Acceptable reference ranges for blood tests, especially for micro-elements in small ruminants, are quite diverse. According to Radostits et al. (2007), the referential value for Se in the goats' blood is from 63.2 to 157.9 $µg\;L^{-1}$ , while, in sheep (Ademi et al., 2017), the referential value for Mn is from 0.1 to 6 $µg\;L^{-1}$ , which indicates the inconsistency in values and the need to expand the testing to a greater number of animals, as well as to include other factors in the testing redesign. Similar conclusions were published by Schweinzer et al. (2017), who reported that all animals in their research were clinically healthy without signs of deficiencies or toxicity with regard to certain tested elements. Puls (1994) also showed different reference values for blood mineral profiles in goats when he highlighted the following values for Ca, P, and Mg and Fe: 8.0–11.0, 4.0–8.0, and 2.0–3.5 mg dL^−1^ and 50–100 $µg\;{\mathrm{dL}}^{-1}$ ), respectively. In our research, the determined average concentrations of some macro-elements and micro-elements (Tables 1 and 2) were similar to those determined in the research of Antunović et al. (2017) carried out on lactating Alpine goats, with the blood being sampled each month, covering the period from the 20th day to the 140th day of lactation. Analysis of the data obtained in this research showed that the concentrations of micro-elements in blood had greater variability, as indicated by the high standard deviations (Cu: 0.50; Mn: 6.04; Fe: 1.04). When compared to our research results, the research on lactating Saanen goats in Italy conducted by Manuelian et al. (2020) determined similar average concentrations of Na at 146.08 mEq L^−1^ (3360 mg L^−1^) and of K at 4.23 mEq L^−1^ (165 mg L^−1^), as well as a higher concentration of Mg (3.23 mg dL^−1^) and lower concentrations of inorganic P at 5.94 mg dL^−1^ and of Ca at 10.04 mg dL^−1^, with a significant influence by the lactation stage throughout the whole lactation period, except in relation to the concentration of K. The above-mentioned authors also determined that the coefficient of variation was less than 10 % for Na at 2.96 % and for Mg at 9.6 % and that the it was from 10 % to 20 % for K at 10.2 % and for Ca at 10.3 %. In the aforementioned research, significant changes in the blood mineral profile were determined depending on the breed of the goats. Compared to Saanen goats in the early (2–4 weeks) and middle stages (5–10 weeks) of lactation, the Na levels were significantly higher in the blood of productively weaker Mediterranean goat breeds (Rosa Mediterranea, Girgentana, and Garganica breeds). Likewise, in the same research, Maltese and Jonica breeds of goats had significantly higher Na concentrations than Saanen goats in the early stage of lactation, while the opposite trend was recorded for K concentrations, which were the lowest in Saanen goats. Thus, the significant effect of goat breed on the blood mineral profile indicates the need to consider the specific breed as one of the criteria for the redesign of blood reference ranges for goats. Compared to our research data, Vilallonga et al. (2012) carried out research on the serum of goats raised on Spanish spring pastures at an altitude of 300 to 600 mm above sea level, and they determined similar average concentrations of Ca and Mg (8.68 and 2.55 mg dL^−1^, respectively) and of Fe (126.0 $µg\;{\mathrm{dL}}^{-1}$ ); a lower concentration of P (6.20 mg dL^−1^); and higher concentrations of Cu, Zn, and Se (169.6, 75.8, and 16.4 $µg\;{\mathrm{dL}}^{-1}$ , respectively). The research of Singh et al. (2022), carried out in India on the blood profile of lactating goats of the Beetal breed, resulted in higher average concentrations of Ca, Fe, and Zn (582.39, 7.62, and 3.59 mg L^−1^, respectively); lower concentrations of Na and K (362.85 and 76.55 mg L^−1^, respectively); and similar concentrations of Cu (1.00 mg L^−1^). In research conducted in Croatia, Samardžija et al. (2011) also stressed the important influence of genotype and/or breed on the concentrations of Ca and P in the blood of goats in puerperium. In comparison to dairy goats (crossbred German Improved Fawn), Boer goats had higher Ca levels and lower P serum levels; however, the German Improved Fawn had an average serum Ca level below the physiological ranges. For this reason, the authors suggested that it would be necessary to define macro-mineral serum levels in small ruminants during puerperium as this could be helpful in the diagnostics of some metabolic disorders in goats.

Analysis of the time of blood sampling in the first third of the lactation period (Tables 3 and 4) confirmed that concentrations of P, K, Mg, Fe, Zn, Mo, and Co were significantly lower in blood on the 90th day compared to on the 30th day of lactation. Concentrations of Ca, Na, Cu, Mn, and Se in the goats' blood did not vary significantly in terms of their dependence on the time of sampling, although they were also lower on the 90th day than on the 30th day of the lactation period. The above-stated findings indicate that the concentrations of minerals in the serum of goats decreased as milk production increased, which is explained by the absorption of minerals from the blood and into the milk. Ahmed et al. (2000) stated that the production of milk during lactation reduced Ca, P, Mg, and K in the blood of Nubian goats and connected such an occurrence with the secretion of these mineral elements from their original sites and into milk. Manuelian et al. (2020) reported that concentrations of Mg and Ca in the goats' serum were the lowest during early lactation when compared to the middle and late stages of lactation for Rossa Mediterranea, Girgentana, and Garganica breeds of goats. When compared to Saanen goats, concentrations of P in the serum of Mediterranean goat breeds were significantly lower in the middle and late stages of lactation. Khaled et al. (1999) determined similar concentrations of Na and K in the blood of dairy goats in the first 3 months of lactation. Potassium is in charge of sustaining the intracellular osmotic pressure, whereas Na is the primary electrolyte in charge of sustaining the volume and osmotic pressure of plasma. When comparing previous studies, Manuelian et al. (2020) concluded that Na and K levels in plasma were less influenced by the stage of lactation. They reported that Ca, P, and Mg concentrations differed between research projects, although Na and K concentrations were similar, and they explained such findings by means of the fact that Ca, P, and Mg were primarily affected by the same homeostatic mechanisms. Compared to the early stage of lactation in Saanen goats (2–4 weeks of milking), the above-mentioned authors determined a significant increase in the concentrations of Mg and Ca in the middle and late stages of lactation (5–10 weeks of milking and 11–30 weeks of milking, respectively) but found no significant changes in the concentrations of Na, K, and inorganic P. Azab and Abdel-Maksoud (1999) determined a decrease in the Fe concentration in the plasma of lactating Baladi goats. Although, in the presented research, no significant decrease in Ca concentration was established in relation to the dependence on the sampling day during the first third of the lactation period, this decrease was still noticeable as lactation progressed. If the research was carried out during the entire lactation period, the authors would expect that these changes in the Ca concentrations in the serum of Alpine goats would have been significant. A faster mobilisation of calcium from the bones may have been produced due to the mammary glands' increased Ca sequestration during lactation (Horst et al., 1997). The investigation of Mezzetti et al. (2024) on Alpine and Saanen goats in Italy confirmed this explanation. Moreover, the decreased plasma concentration of P is compatible with the quicker fluid redistribution caused by milk synthesis (Cappai et al., 2019). The same was reported by Allaoua and Mahdi (2018) in research conducted in Algeria on Arabian goats at 3 to 16 weeks of lactation, within which they confirmed that Ca in plasma increased significantly at the end of the lactation period, which was correlated with a decreased need for calcium due to reduced milk secretion in the late stage of lactation or with increased intestinal absorption of Ca. Furthermore, Tosto et al. (2021) investigated dairy goats in Italy and found that, during the first few weeks of lactation, Ca concentrations in the goats' blood tend to decrease due to increased milk production. While evaluating goats during the transition period, Tharwat et al. (2013) observed a 20 % decrease in total calcium concentration during the kidding week.

Many significant correlations between concentrations of minerals in the blood of Alpine goats during the first third of the lactation period (Table 5) confirm their mutual dependence and intertwining in the animal metabolism. Similar results were obtained by Antunović et al. (2021) in their tests on Travnik Pramenka sheep blood. In comparison with the presented research, for the period from 30 to 120 d of lactation, the mentioned authors also determined significant positive correlations between macro-elements in the blood of sheep ( Ca:P , Ca:K , K:Na , K:Mg , P:Na ), between macro-elements and micro-elements ( Na:Zn , Na:Se ), and between micro-elements ( Fe:Zn ). In a study carried out on cows during different stages of lactation, Abd-El Naser et al. (2014) determined positive correlations between Ca:K in the cows' blood, and El Zubeir et al. (2005) also found such a positive correlation for Cu:Z in the blood of lactating cows. Moreover, Fadlalla et al. (2020) also published similar significantly positive correlations in lactating cows' serum for Ca:P , Ca:Mg , P:Mg , P:Na , Na:K , and Na:Mg . Confirmed correlations may help in monitoring the effect of nutrition on blood mineral profiles and in indicating if possible supplementation is needed, or they may provide information on animals' health statuses during the lactation period.

## 5 Conclusion

The results of blood tests obtained in this research identified mineral profiles in the blood of lactating French Alpine goats and contributed to the widening of the knowledge on the metabolic adaptation of the breed in light of their possible spreading around the world. Currently, there are many different reference ranges for the determination of animals' blood mineral profiles, i.e. concentrations of macro-elements (Ca, P, K, Na, Mg) and micro-elements (Fe, Zn, Cu, Mn, Se, Mo, Co), and so it is recommended that one review the ranges applicable to goats' blood. With respect to goat breeds, reference ranges should be unified for the Alpine goat breed to include physiological factors, such as lactation. The definition of a unified reference range should be accompanied by validation of quality laboratory methods for the analysis of blood mineral profiles.

## Data Availability

The data are available from the corresponding author upon reasonable request.
